# [Retracted] Inhibition of neddylation modification by MLN4924 sensitizes hepatocellular carcinoma cells to sorafenib

**DOI:** 10.3892/or.2025.8866

**Published:** 2025-01-13

**Authors:** Zelong Yang, Jie Zhang, Xiaotong Lin, Di Wu, Guixi Li, Chunlian Zhong, Lei Fang, Peng Jiang, Liangyu Yin, Leida Zhang, Ping Bie, Chuan-Ming Xie

Oncol Rep 41: 3257–3269, 2019; DOI: 10.3892/or.2019.7098

Following the publication of the above article, the authors noticed that they had inadvertently included a duplication of the same data in Fig. 3C, portraying colony formation experiments, where the results from differently performed experiments were intended to have been shown, and requested that a corrigendum be published to present the data in this figure accurately.

Having investigated this matter in the Editorial Office, however, additional panels of overlapping data were identified, comparing between Figs. 2 and 3; moreover, a pair of overlapping data panels were also identified examining the Transwell migration assay data in Fig. 5A. The Editor of *Oncology Reports* has considered the authors' request to publish a corrigendum, but has decided to decline this request on account of the additional errors that have been identified; rather, the article is to be be retracted from the Journal on the basis of an overall lack of confidence in the presented data. Upon receiving this decision from the Editor, the authors did not provide a satisfactory reply. The Editor apologizes to the readership of the Journal for any inconvenience caused.

